# The Current Situation and Influencing Factors of Job Stress Among Frontline Nurses Assisting in Wuhan in Fighting COVID-19

**DOI:** 10.3389/fpubh.2020.579866

**Published:** 2020-10-22

**Authors:** Yufang Zhan, Shuang Ma, Xiangdong Jian, Yingjuan Cao, Xiangqiao Zhan

**Affiliations:** ^1^Department of Neurology, Qilu Hospital, Shandong University, Jinan, China; ^2^School of Nursing, Cheeloo College of Medicine, Shandong University, Jinan, China; ^3^Department of Poisoning and Occupational Diseases, Emergency, Qilu Hospital, Shandong University, Jinan, China; ^4^Department of Nursing, Qilu Hospital, Shandong University, Jinan, China; ^5^Department of Cardial Surgery, Qilu Hospital, Shandong University, Jinan, China

**Keywords:** a cross-sectional survey, COVID-19, epidemic, job stress, nurses

## Abstract

**Background:** The coronavirus disease 2019 (COVID-19) pandemic continues to pose an unprecedented threat and challenge to public health around the world. Lacking sufficient human resources, nurses are required to undertake an increased workload at the clinical frontline of this epidemic. Additionally, nurses are at a high risk due to their working within close proximity to COVID-19 patients. As a result, they experience increased job stress.

**Objective:** To explore the current situation and influencing factors of job stress among clinical first-line nurses fighting COVID-19.

**Methods:** A convenience sampling method was used to conduct a questionnaire survey with 110 nurses who were on the clinical frontline of the COVID-19 epidemic in a hospital in Wuhan.

**Results:** The job stress scores of clinical nurses on the frontline of the COVID-19 epidemic were collected (91.42 ± 26.09); the dimensions of work environment and resources as well as workload and time pressure were ranked first and second, respectively. The results of a multiple stepwise regression analysis showed that working hours per day, service years, number of night shifts per week, and level of academic qualification were the main factors affecting the job stress levels of nursing staff.

**Conclusions:** The job stress of nurses on the clinical frontline of the COVID-19 epidemic was found to be at a medium level. Nursing leaders and hospital managers should pay attention to the impact of job stress on frontline nurses, while taking positive and effective measures aimed at eliminating the source of nursing work pressures to stabilize their nursing teams and promote their work in the fight against this epidemic.

## Introduction

The coronavirus disease 2019 (COVID-19), which originated in Wuhan, Hubei Province, China, at the end of 2019, has become a major public health problem in not only China but also on a global scale (1, 2). On January 20, 2020, the National Health Commission of China announced that COVID-19 would be included in the management of Class B infectious diseases, and prevention and control measures for Class A infectious diseases were then adopted (3). With the continuous spread of this epidemic, a large number of both confirmed and suspected patients have gradually flooded into various major hospitals, significantly increasing the workload and pressures of clinical frontline nurses.

Due to a lack of human resources, nurses are required to undertake a significant amount of work at the clinical frontline of this epidemic. They do not only perform therapeutic work for patients but also provide life care, basic care, and psychosocial care (4). Moreover, they are involved in developing and opening new wards and are responsible for the disposal of medical waste among other responsibilities. To protect themselves, nurses need to wear personal protective equipment (PPE)—including goggles, N95 masks, and protective suits—while at work. However, using PPE over long periods of time can be exhausting. For example, when wearing a N95 mask, nurses need to speak loudly to ensure that their patients can hear them.

Job stress refers to an individual's response to the characteristics of the work environment, which appears to be threatening and is caused by the individual's perceptions of the imbalance between job demands and their abilities to cope with those demands (5, 6). There are many sources of job stress for nurses. As has been established, increasing one's workload can aggravate a person's stress levels. Particularly during this unique period, nurses are more stressed than ever as they are faced with a highly contagious disease and unfamiliar work environments and patterns (7). Moderate job stress is conducive in stimulating the potential of nurses (8, 9). However, some studies have shown that extreme job stress caused by excessive job-related demands (e.g., increasing workload, inadequate work environment) is more likely to lead to burnout (10, 11). Simultaneously, working excessively and compulsively may affect well-being at work and increase the risk of burnout (12–16). All these affect their overall work quality, which is not conducive to the stability of a hospital's nursing team (17, 18). One study found that the cumulative effects of repeated exposure to stressors results in poorer mental health and functioning (19). Our study is therefore of great significance.

Our study had two primary objectives. The first was to provide a reference for hospital managers to take effective and practical measures toward protecting the health and wellness of their nursing staff. The second was to reduce the physical and mental harm experienced by frontline nurses from various sources of stress to stabilize hospitals' nursing teams and promote their work in combating COVID-19.

## Methods

### Study Population and Design

Using convenient sampling, nurses from Shandong, who were supporting a Wuhan hospital in combatting the COVID-19 epidemic during the period between February 9 and March 31, 2020, were selected for this survey. The inclusion criteria were as follows: nurses from Shandong who were involved in treating COVID-19 patients, those who entered the clinical frontline to participate in rescue work, and those who volunteered to participate in this study.

### Measurements

#### Demographic Details Form

Based on our literature review and expert consultation (20–25), we created self-made general information and Demographic questionnaires, including questions on sex, age, marital status, education, professional qualifications, service years, working hours per day, and number of working night shifts per week since the outbreak began.

#### Nurse Job Stressors Scale

The nurse job stressors scale was developed by Li and Liu (24), who based it on the two most commonly used foreign nurse job stressors scales (26, 27). According to the Chinese perspective, they redesigned the nurse job stressors scale and consulted nursing experts in the United States, Thailand, and China to revise the Chinese Nurses' Job Stressors scale. This scale has good reliability and validity (25), with a Cronbach's α value of 0.94. The scale has 35 entries divided across five dimensions: nursing profession and work, time and workload allocation, working environment and resources, patient care, and management and interpersonal relationships. A Likert 4-point rating scale was adopted to assess each item, from “no pressure” to “a lot of pressure,” with each comprising 1–4 points that are totaled, with a higher total score representing a greater level of pressure experienced. In our study, scores of 35–70 were classified as mild stress, those of 71–105 as moderate stress, and those of 106–140 as severe stress.

### Survey Methods and Data Collection

Online surveys (via a questionnaire website platform) were sent out to the participants. The participants completed the questionnaires using either a computer or a smartphone that could open the website link or scan the quick response code. The investigator initially explained, in detail, the purpose and significance of this study, how participants' anonymity and confidentiality would be ensured, as well as other relevant information. After the participants signed the informed consent form, they scanned the code and entered their responses into the answer interface. As of March 31, 2020, 110 frontline clinical nurses were invited to participate in this study, with a response rate of 100%.

### Statistical Analyses

Counting data were expressed as frequencies and percentages, with the measurement data being expressed as $\bar{\chi }$ ± s. Comparisons between the two groups were performed using two independent-sample *t*-tests, with comparisons between multiple groups being performed using a single-factor ANOVA. A multiple stepwise linear regression analysis was then performed to conduct multivariate analysis.

## Results

### Basic Information

The distribution of demographic data for the 110 frontline clinical nurses working during the COVID-19 epidemic is shown in Table 1.

**Table 1:** Demographic characteristics of study participants (*n* = 110).

**Variable**		**Number (*n*)**	**Percentage (%)**
Gender	Male	25	22.7
	Female	85	77.3
Age	<30	47	42.7
	30–34	32	29.1
	35–39	17	15.5
	≥40	14	12.7
Marital status	Unmarried	26	23.6
	Married	81	73.6
	Divorced or widowed	3	2.8
Level of academic qualification	Junior college or below	7	6.4
	Undergraduate	95	86.3
	Master or above	8	7.3
Professional qualification	Primary	63	57.3
	Intermediate or above	47	42.7
Service years	≤5 years	25	22.7
	6–10 years	37	33.7
	11–20 years	26	23.6
	≥21 years	22	20.0
Working hours per day	≤6 h	76	69.1
	7–10 h	28	25.5
	≥11 h	6	5.4
Number of night shifts per week	≤2	40	36.4
	3–4	61	55.5
	≥5	9	8.1

### Job Stress of Nurses

The average job stress score of the 110 frontline nurses assisting in combating the COVID-19 epidemic was 91.42 ± 26.09, which represents a moderate stress level. The specific scores of each dimension are shown in Table 2.

**Table 2:** Job stress scores of front-line nurses assisting in combating the COVID-19 epidemic ($\bar{\chi }$ ± s, *n* = 110).

**Dimension**	**Score**		**Rank**
	**Dimension**	**Entry**	
Nursing profession and work	18.43 ± 4.51	2.63 ± 0.64	3
Workload and time pressure	15.32 ± 5.36	3.06 ± 1.07	2
Resource and environmental	9.25 ± 3.33	3.08 ± 1.11	1
Patient care and interaction	28.21 ± 6.82	2.56 ± 0.62	4
Interpersonal relationships and management	20.21 ± 6.07	2.25 ± 0.67	5
Total score	91.42 ± 26.09	2.61 ± 0.75	

### Single-Factor Analysis of the Job Stress of Nurses

Our results showed that gender, level of academic qualification, professional qualification, service years, working hours per day, and number of night shifts per week all impacted frontline nurses' job stress scores (*p* < 0.05), as shown in Table 3.

**Table 3:** Single-factor analysis of the job stress of front-line nurses ($\bar{\chi }$ ± s, *n* = 110).

**Variable**		**Total points**	**Statistics**	***p*-value**
Gender	Male	83.99 ± 24.81	*t* = −2.534	0.013
	Female	94.79 ± 26.30		
Age	<30	87.41 ± 25.34	*F* = 1.168	0.204
	30–34	91.19 ± 26.68		
	35–39	97.84 ± 24.99		
	≥40	93.65 ± 23.81		
Marital status	Unmarried	87.10 ± 24.60	*F* = 0.958	0.388
	Married	93.29 ± 26.74		
	Divorced or widowed	96.07 ± 21.75		
Level of academic qualification	Junior college or below	72.36 ± 23.17	*F* = 6.067	0.003
	Undergraduate	92.09 ± 26.44		
	Master or above	106.04 ± 21.55		
Professional qualification	Primary	88.09 ± 24.67	*t* = −2.220	0.029
	Intermediate or above	96.45 ± 26.79		
Service years	≤5 years	83.85 ± 25.34	*F* = 2.995	0.035
	6-10 years	90.65 ± 24.43		
	11–20 years	97.02 ± 25.36		
	≥21 years	99.04 ± 17.87		
Working hours per day	≤6 h	88.64 ± 25.42	*F* = 4.354	0.016
	7–10 h	93.98 ± 21.85		
	≥11 h	105.43 ± 27.26		
Number of night shifts per week	≤2	87.35 ± 25.96	*F* = 4.484	0.014
	3–4	93.48 ± 23.96		
	≥5	99.70 ± 27.32		

### Multiple-Factor Analysis of the Job Stress of Nurses

The job stress scores of the frontline nurses were the dependent variable, with the single-factor analysis of their stress load with statistically significant titles then being used as the independent variables for the stepwise multiple linear regression analysis. The results showed that working hours per day, service years, number of night shifts per week, and level of academic qualification were the main factors influencing the job stress levels of nurses assisting in the fight against COVID-19, which explains 65.3% of the total variance, as shown in Table 4.

**Table 4:** Multiple-factor analysis of the job stress of front-line nurses.

**Dependent variable**	**Regression coefficient**	***SE***	**Standardized regression coefficient**	***t*-value**	***p*-value**
Constant	10.364	2.651		2.835	0.006
Working hours per day	2.085	0.498	0.332	4.195	0.000
Service years	0.776	0.223	0.283	3.523	0.001
Number of night shifts per week	2.403	0.652	0.296	3.687	0.000
Level of academic qualification	7.744	3.209	0.161	2.415	0.018

*R = 0.807, R^2^ = 0.653, F = 39.886, P < 0.01*.

## Discussion

### Job Stress Analysis of Nurses Assisting in Wuhan in Treating COVID-19

The job stress of nurses refers to their mental and physical stress reactions and states caused by the cognitive requirements of the subjective and objective conditions in their workplace that exceed their adaptability (28). Our results found that the average total job stress score of the participating 110 frontline clinical nurses was 91.42 ± 26.09, which was moderately high in general and was higher than the survey results of ordinary nursing staff as reported by Gao et al. (29). The top two scores in each dimension were those of work resource and environment, and workload and time pressure, which was inconsistent with the results of the general nursing staff (29), and consistent with the results of Lin et al. in the infection department nursing staff (30). As an emerging infectious disease, COVID-19 has made it difficult for many hospitals to meet the needs of frontline clinical nurses in both their working environments and medical facilities. Many hospital wards used in this epidemic are converted from the general wards. Nurses work in relatively closed spaces, with inadequate medical facilities, and often in highly infectious environments for long periods of time, which is particularly harmful during this epidemic (31). Nurses assisting in Wuhan in the fight against COVID-19 have a heavier workload and greater level of responsibility. In addition to taking care of patients and avoiding infection as far as possible, they also need to strengthen their own professional knowledge, participate in various trainings that provide knowledge on COVID-19, publicize that same knowledge, and report on the epidemic to their patients. This makes the job of these frontline clinical caregivers more stressful than that of other general nurses. Therefore, we suggest that hospital managers should attach greater prioritization to the working pressures of frontline nurses and take active and effective measures to improve their working environments by equipping them with sufficient materials, reasonably arranging their working hours, and appropriately adjusting the proportions of staff members.

### Factors Influencing the Job Stress of Nurses Assisting in Wuhan in the Fight Against COVID-19

Our results showed that working hours per day, service years, number of night shifts per week, and level of academic qualification were the main factors influencing the job stress of nurses assisting in the fight against COVID-19.

(1) Working hours per day: This study found that the longer the working hours, the more stressful the job of nurses was. Since the outbreak began in Wuhan, there has been a severe shortage of frontline nurses, who are often overworked as it is. According to a survey conducted by Zhang et al. (32) on the preferred work hours per shift among frontline nurses during the COVID-19 epidemic, 60.55% of them regarded 4 h as the preferred number for each work shift, while the actual shift length far exceeded these preferred hours. The specific nature of COVID-19 increases the risk of occupational exposure for nurses, requiring them to wear protective suits, N95 masks, goggles, rubber gloves, etc. Multiple protective measures result in increased inconveniences for nursing staff. In addition, the complicated conditions of COVID-19 patients mean that most are unable to take care of themselves, let alone be accompanied by their families. Thus, in addition to completing the treatment work and basic care services while on duty, frontline nurses also need to conduct psychotherapeutic communications with the patients to relieve their tension and anxiety. Working in a closed environment for too long can lead to sweating, shortness of breath, irritability, an inability to concentrate, and various negative emotions, which can then lead to increased job stress levels.

(2) Number of night shifts per week: Our results show that the higher the number of night shifts per week, the higher the stress levels of nurses. This may be due to the shortage of frontline nursing staff resulting in more frequent night shifts, forcing nurses to work in a high-intensity state, which then causes biological clock disorder and changes in their endocrine systems (33). Research (34) shows that job stress not only negatively affects nurses' physical health, but also impairs their overall sleep quality. Lack of sleep in any form can lead to decreased concentration, anxiety, irritability, confusion, depression, emotional numbing, or indifference (35). In addition, various studies have shown that working night shifts tends to increase nurses' emotional exhaustion and work fatigue (36). Simultaneously, night nurses must deal with medical emergencies and other strenuous scenarios (37), which causes the frontline night shift nursing staff to experience an increased sense of job pressure.

(3) Level of academic qualification and service years: Our results showed that nurses with higher seniority and educational achievements also had higher job stress levels than those of lower seniority and education level, which is consistent with the results reported by Ouhuan et al. (38). This may be because nurses with higher seniority and educational levels have accumulated more practical clinical experiences. They are often more competent in clinical management and teaching in the context of frontline nursing work. They tend to undertake more responsibilities, train, and guide junior nurses, and have a higher sense of responsibility for the patients, thus generating greater job pressure.

### Implications for Nursing Managers

Nursing managers should pay attention to the impact of job stress on frontline nurses and take active and effective measures aimed at eliminating the sources of nursing pressures to stabilize their nursing teams and assist them in the fight against the COVID-19 epidemic. Research shows that optimizing human resource management (39) and employee assistance plans (40) can allow organizations to effectively utilize and develop their human resources. Hospitals can create an adaptive working environment through incentive management, hierarchical training, group management, and optimization of shift scheduling, which would then help to relieve the job stress of frontline nurses in the fight against COVID-19.

## Limitations

Our study has the following limitations. First, through convenient sampling, only nurses from Shandong, who were supporting Wuhan in combatting the COVID-19 epidemic, were selected for this survey. Hence, the generalizability of our findings may be limited. Second, due to the cross-sectional design, certain limitations exist in our study. We only assessed job stress at a particular time, without longitudinal observation. Thus, we hope that future research will involve a longitudinal observation of the participants. Third, unfortunately, we only evaluated the participants and did not intervene. Hence, an intervention for participants in future research is necessary. Finally, in addition to the factors included in our study, there may be others that affect the job stress of nurses.

## Conclusion

COVID-19 is a huge public health challenge worldwide. During the COVID-19 epidemic, the job stress of nurses is worthy of attention. In our study, the job stress of nurses assisting in Wuhan in treating COVID-19 was generally higher. Working hours per day, service years, number of night shifts per week, and level of academic qualification were the main factors influencing the job stress of nurses. Meanwhile, we shared some methods to relieve the stress that may provide a reference for nurses in other countries to adjust job stress.

## Data Availability Statement

The raw data supporting the conclusions of this article will be made available by the authors, without undue reservation.

## Ethics Statement

The studies involving human participants were reviewed and approved by Ethics Committee of Qilu Hospital, Shandong University. The patients/participants provided their written informed consent to participate in this study. Written informed consent was obtained from the individual(s) for the publication of any potentially identifiable images or data included in this article.

## Author Contributions

YZ and YC conceived the study. SM created and performed the literature search strategy. YZ and XZ collected questionnaires. YZ and SM performed the data extraction. YZ wrote the original draft. XJ and YC reviewed the manuscript and supervised the process. All authors read and approved the final manuscript.

## Conflict of Interest

The authors declare that the research was conducted in the absence of any commercial or financial relationships that could be construed as a potential conflict of interest.
